# From Snapshots to Development: Identifying the Gaps in the Development of Stem Cell‐based Embryo Models along the Embryonic Timeline

**DOI:** 10.1002/advs.202004250

**Published:** 2021-03-02

**Authors:** Vinidhra Shankar, Clemens van Blitterswijk, Erik Vrij, Stefan Giselbrecht

**Affiliations:** ^1^ Maastricht University Universiteitssingel 40 Maastricht 6229 ER The Netherlands

**Keywords:** bioengineering, blastoids, embryo development, extraembryonic tissues, stem cell‐based models

## Abstract

In recent years, stem cell‐based models that reconstruct mouse and human embryogenesis have gained significant traction due to their near‐physiological similarity to natural embryos. Embryo models can be generated in large numbers, provide accessibility to a variety of experimental tools such as genetic and chemical manipulation, and confer compatibility with automated readouts, which permits exciting experimental avenues for exploring the genetic and molecular principles of self‐organization, development, and disease. However, the current embryo models recapitulate only snapshots within the continuum of embryonic development, allowing the progression of the embryonic tissues along a specific direction. Hence, to fully exploit the potential of stem cell‐based embryo models, multiple important gaps in the developmental landscape need to be covered. These include recapitulating the lesser‐explored interactions between embryonic and extraembryonic tissues such as the yolk sac, placenta, and the umbilical cord; spatial and temporal organization of tissues; and the anterior patterning of embryonic development. Here, it is detailed how combinations of stem cells and versatile bioengineering technologies can help in addressing these gaps and thereby extend the implications of embryo models in the fields of cell biology, development, and regenerative medicine.

## Graphical abstract

This progress report aims to highlight some of the potential gaps in the developmental timeline of synthetic stem cell‐based embryo models relative to natural embryogenesis and provides innovative bioengineering solutions to understand and eventually improve the modeling of mammalian embryo development in the future.

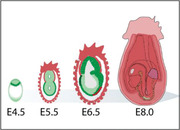

## 1 Introduction

The natural embryo has been the archetypical model for studying mammalian self‐organization and has greatly contributed to our understanding of core biological processes, such as pluripotency,^[^ ^1^, ^2^ ^]^ differentiation, and development,^[^ ^3^, ^4^, ^5^ ^]^ and far‐reaching applications including in vitro fertilization, cellular reprogramming, and genetic modifications.^[^ ^6^ ^]^ In vitro development of mammalian embryo models is a powerful means to investigate specific morphological events during development. Over the years, studies on vertebrate embryos have helped in understanding crucial events such as cell‐fate specification,^[^ ^7^, ^8^ ^]^ lineage commitment,^[^ ^9^ ^]^ tissue patterning,^[^ ^10^, ^11^ ^]^ and multicellular morphogenetic events.^[^ ^12^, ^13^ ^]^ Although the use of natural embryos has been instrumental for elucidating mechanisms of embryonic development, they too entail difficulties related to accessibility, size, freedom of perturbation, and access to specific developmental time windows. Notably, their use as experimental models comes with ethical concerns even more so beyond implantation. With the advent of versatile bioengineering technologies, several 3D stem cell‐based embryo models have made the spotlight as a tool to deepen our understanding of the processes governing development and provide a more realistic approach to compare these scenarios with reduced ethical implications. Exploiting the inherent self‐organizing capacity of pluripotent stem cells (PSCs) and extraembryonic stem cells coupled with the timely manipulation of microenvironmental signals permits recapitulating development in‐a‐dish. In contrast to embryos, embryo models are permissive to physically and genetically disentangling different tissue compartments to accurately study their reciprocal roles in development. In addition, the large‐scale formation of embryo models enables high‐throughput experimentation.

**Figure 1:**
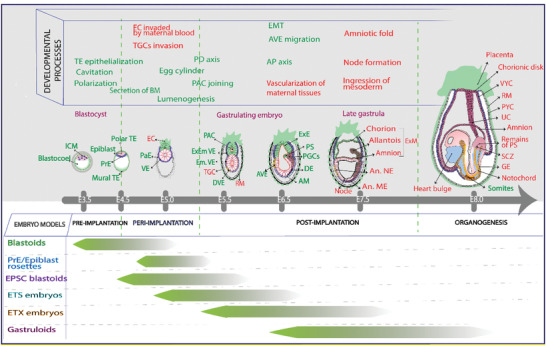
Schematic representing the current mouse embryo models along the timeline of mouse embryonic development. The developmental processes and tissues that have been generated in the in vitro embryo models are shown in green and the text in red highlights the gaps that are yet to be resolved. ICM, inner cell mass; TE, trophectoderm; PrE, primitive endoderm; EC, ectoplacental cone; PaE, parietal endoderm; VE, visceral endoderm; PAC, pro‐amniotic cavity; TGC, trophoblast giant cells; DVE, dorsal VE; AVE, anterior VE; RM, Reichert's membrane; ExE, extraembryonic ectoderm; PS, primitive streak; PGC, primordial germ cells; DE, definitive endoderm; AM, axial mesoderm; ExM, extraembryonic mesoderm; An. NE, anterior neurectoderm; An. ME, anterior mesendoderm; VYC, visceral yolk sac; PYC, parietal yolk sac; UC, umbilical cord; SCZ, stem cell zone; GE, gut endoderm; EMT, epithelial‐mesenchymal transition.

**Figure 2:**
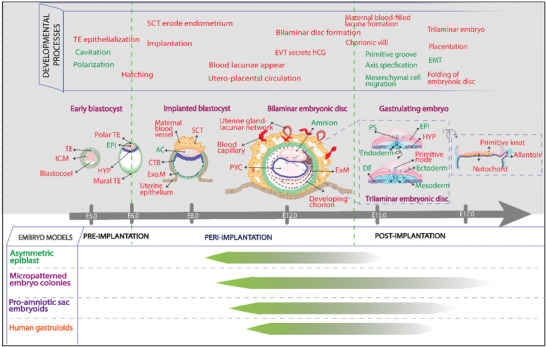
Schematic representing the current human embryo models along the timeline of human embryonic development. The developmental processes and tissues that have been generated in the in vitro embryo models are shown in green and the text in red highlights the gaps that are yet to be resolved. ICM, inner cell mass; TE, trophectoderm; PrE, primitive endoderm; EPI, epiblast; HYP, hypoblast; SCT, syncytiotrophoblast; CTB, cytotrophoblast; Exo.M, exocoelmic membrane; AC, amniotic cavity; ExM, extraembryonic mesoderm; PS, primitive streak; PYC, primitive yolk sac; DE, definitive endoderm; EVT, extravillous trophoblast; hCG, human chorionic gonadotropin; EMT, epithelial‐mesenchymal transition.

Despite serving as an important tool to observe early embryogenesis, there are still some primary gaps in modeling embryogenesis with respect to the developmental timeline. Stem cell‐based embryo models have been derived for mouse and human systems (**Figures** **1** and **2**), both of which achieve specific morphogenetic events and tissue types when exposed to a carefully selected medium cocktail. Each of these embryo models require very specific culture parameters, such as initial cell numbers, e.g., around 300 embryonic stem cells (ESCs) are required for generating gastruloids^[^ ^14^ ^]^ and around 4–10 ESCs for generating blastoids^[^ ^15^ ^]^, or the type of platform used to culture these multicellular constructs. Such intricate features dictate the developmental potential of the 2D or 3D systems.

However, there are some significant heterogeneities in the culture of these embryo models which is evident with the low efficiency of production and variation between different strains of cell lines used. Further optimization of the culture conditions may be required to achieve more robust developmental potential. Further, the current embryo models recapitulate only snapshots within the window of embryonic development. This is more evident in the human system where there is comparatively lesser knowledge on the development beyond implantation. The current human models can acquire only limited embryonic patterning and specification, while most of the spatio‐temporal confinement of the structures and the extra‐embryonic derivatives are still lacking. Hence, there is a need for identifying and bridging the gaps to attain additional embryo models spanning a wider range of the morphogenetic spectrum in early development.

This progress report highlights some of the most important gaps along the timeline of the mouse (Figure 1) and human (Figure 2) embryonic development and provides potential strategies for filling these in the future with the aid of micro‐ and bioengineering approaches. The report further provides valuable insights on how to extend the existing models further, to be a guideline for future studies, and to develop new bona fide models to finally better reproduce the natural embryonic development with key spatial and temporal features.

## 2 In Vitro Embryo Models

### 2.1 Embryoid Bodies

Embryonic stem cells (ESCs) are traditionally derived from the blastocyst stage of the embryo, although ESCs from the morula or earlier stages have also been derived in the past. ESCs have a remarkable capacity to self‐organize into 3D structures, called embryoid bodies (EBs),^[^ ^16^ ^]^ that confer spontaneous differentiation and patterning events reminiscent of embryo development (Table **1**). EBs occupy a very significant place as a model to study mammalian embryogenesis through the well‐established expansion and differentiation protocols that can provide valuable information on the plasticity of ESCs and their ability to be manipulated by modulating their surrounding microenvironment.

**Table 1:** The current mouse embryo models with the platform in which they are cultured, the major finding using that model, and the morphological stage of mouse development they represent

Embryo model	Culture platform	Significant finding	Stage of embryonic development they represent
Embryoid bodies (mESCs)	Hanging drops, suspension culture,^[^ ^31^ ^]^ microwell plate, ^[^ ^32^ ^]^ or hydrogels^[^ ^33^, ^34^ ^]^	– Directed differentiation of EBs produces endoderm, mesoderm, neural and cardiomyocytes ^[^ ^17^ ^]^	
Mouse peri‐implantation epiblast rosettes	Agarose hydrogels^[^ ^22^ ^]^	– Basement membrane secretion cues for lumenogenesis and apico‐basal polarization in epiblast rosettes through *β*‐integrin receptors – ESCs exposed to a chemical cocktail of Wnt, Fgf4, RA and cAMP is sufficient to induce co‐development of epiblast and primitive endoderm which progresses to post‐implantation morphogenesis ^[^ ^22^ ^]^	E4.0–4.5 to E4.5‐5
Trophospheres (TSCs)	Hanging drop, ultra‐low attachment culture dishes, suspension^[^ ^35^ ^]^	– BMP4 and Nodal promote epithelialization and proliferation in TSCs and increase cavitation rate and size of cavities in trophospheres ^[^ ^15^ ^]^ – TSCs readily form trophospheres at 16% O_2_, although 3% O_2_ led to differentiated cell types → parietal TGC ^[^ ^35^ ^]^ – *Fgf4* exposure generated different morphologies ^[^ ^15^ ^]^	E3.5–E5.0
Mouse blastoids (mESCs+mTSCs)	Agarose hydrogel microwells^[^ ^15^ ^]^	– cAMP and Wnt activation induces cavitation in the blastoids forming a polarized ICM within a TE shell^[^ ^15^ ^]^ – Nodal signals from epiblast promote trophoblast proliferation, self‐renewal, and epithelialization^[^ ^15^ ^]^ – An induction cocktail of Wnt, Fgf4, RA, and cAMP promotes the formation of PrE in blastoids^[^ ^15^ ^]^	E3.5–E4.5
Mouse EPS blastoids (mEPSCs)	Inverted pyramidal microwells ^[^ ^25^ ^]^	– EPSCs self‐organized to generate 8‐cell‐like embryos expressing cell adhesion and tight junction proteins and further into morula‐like structures with an apico‐basal polarity^[^ ^25^ ^]^ – Specification of TE and ICM specification occurs through Hippo‐YAP signaling^[^ ^25^ ^]^ – EPSC blastoids develop post‐implantation epiblast rosettes with a lumen through signals from the ExE‐like TE compartment and VE surrounding the epiblast^[^ ^25^ ^]^	E2.0–E5.5
Mouse ETS embryo (mESCs+mTSCs)	3D ECM Matrix drop → matrigel on µ‐plates (Ibidi) ^[^ ^23^ ^]^	– ESCs and TSC clumps interact to give rise to polarization and lumenogenesis in the epiblast when embedded in an ECM matrix – Matrigel takes the role of VE in the secretion of the basement membrane and promotes rosette formation and lumenogenesis	E4.75–E6.5
Mouse EPS embryos (mEPSCs+mTSCs) ^[^ ^24^ ^]^	Inverted pyramidal microwells (AggreWell) ^[^ ^24^ ^]^	– EPS blastoids develop better cystic structures in hypoxic conditions of 5% O2 compared to normal conditions^[^ ^24^ ^]^ – PaE‐like cells distributed along the TE migrating from PrE resembled an E4.5 embryo^[^ ^24^ ^]^ – The PaE‐like cells were validated by the group of cells expressing Snail, Follistatin, Vimentin, Grem2, and Stra6 on single‐cell transcriptomics data^[^ ^24^ ^]^	E3.5–E5.0
ETX embryo (ES+TS+XEN) ^[^ ^28^, ^35^ ^]^	Inverted pyramidal microwells (AggreWell) Single‐cell suspensions of 3 cell types seeded together ^[^ ^28^ ^]^ Non‐adherent suspension shaking culture^[^ ^35^ ^]^	– Self‐organization of XEN cells along with ES and TS cells, fulfilled the requirement for spontaneously inducing an anterior symmetry breaking event with AVE specification and epithelial‐mesenchymal transition (EMT)^[^ ^28^ ^]^	E5.25–E7.0
Mouse gastruloids (ESC)	Hanging drop model, 96‐well plate, gelatin‐coated tissue culture flasks^[^ ^14^ ^]^ Shaking culture^[^ ^36^ ^]^	– The time of exposure, the combination of signaling regulators, and the size of the aggregates determine the nature of morphogenesis in gastruloids, displaying the dynamicity of in vivo embryos^[^ ^14^ ^]^ – Timely exposure to a Wnt agonist induces gastruloids to self‐organize in vitro into some of the posterior‐specific tissue types like neural, mesoderm, and endoderm‐like cells^[^ ^36^ ^]^	E5.5‐6 to E7.5

EBs have been explored for their potential to specify extraembryonic endoderm and the three embryonic germ layers, namely endoderm, mesoderm, and ectoderm (reviewed in ref. ^[^ ^16^ ^]^), and undergo specific morphogenetic events, such as the establishment of the anterior/posterior axis,^[^ ^17^ ^]^ germ layer specification,^[^ ^14^ ^]^ contracting neural progenitors ^[^ ^18^ ^]^ and cardiomyocytes,^[^ ^19^ ^]^ in response to specific morphogen gradients, such as Wnt,^[^ ^17^ ^]^ BMP,^[^ ^20^ ^]^ or a combination of Wnt and Nodal (Table 1). Although the manipulation of EBs to generate differentiated tissues does not truly capture natural development, these models have enabled the visualization and analysis of a rather complex and closely linked series of events. Of note, as EBs are generally differentiating in chemically‐undefined conditions with a low level of control and reproducibility, studying the molecular and physical underpinnings can be challenging.

### 2.2 Mouse Peri‐ and Post‐Implantation Embryo Models

Mammalian life begins with a series of largely conserved cell fate decisions that prepare the embryo to give rise to the embryonic part, the inner cell mass (ICM), and the extraembryonic part, the trophectoderm (TE).^[^ ^21^ ^]^ A further round of asymmetric divisions in the ICM induces a third type of cells, called the primitive endoderm (PrE) that later forms the yolk sac and helps to pattern the embryo. Peri‐implantation stage is when the embryo implants into the mother's endometrium followed by morphogenesis in the fetal as well as maternal tissues to establish conception. This is the stage when the mouse and human systems diverge in their developmental events. With the increasing knowledge on early developmental processes, 3D embryo models have been developed using stem cells in specific engineered microenvironments, which closely resemble the early mammalian embryos.

#### 2.2.1 Mouse Blastoids

Recently, it has been shown that the combination of mouse embryonic stem cells (mESCs) and trophoblast stem cells (mTSCs) within agarose hydrogel microwells exposed to the signaling regulators Chir (β‐catenin/Wnt activation), cAMP, Fgf4, Il11 (Stat3), and Tgfβ1 can self‐organize into a 3D structure reminiscent of the pre‐implantation blastocyst, called blastoid. Blastoids contain an inner cell mass‐like cell cluster engulfed by an expanded fluid‐filled trophectoderm epithelium and display gene expression profiles comparable to natural embryos.^[^ ^15^ ^]^ A combination of single‐cell RNA sequencing and large‐scale generation of blastoids were used to identify and experimentally pinpoint, respectively, Bmp4/Nodal as inductive signals originating from the inner embryonic cells that promote the self‐renewal, proliferation, and epithelialization of the trophectoderm. The embryonic cells drive TE expansion to form the blastocoel cavity, simultaneously polarizing the ICM to one side.

Modulating the signaling requirement in the blastoids using small molecules including Fgf4, retinoic acid, Chir and cAMP gave rise to PrE‐like cells flanking the ICM at the embryonic side.^[^ ^22^ ^]^ Further culture of blastoids promoted the morphogenesis of the pluripotent epiblast into post‐implantation stage rosette‐like structures enveloped by an extraembryonic endoderm layer. Blastoids occupy an important place in the embryo modeling landscape mimicking the crucial pre‐ to post‐implantation transition by promoting the timely cross‐talk between niches and its visualization on a large‐scale with the use of genetic tools, thereby allowing the study of signaling regulations between individual compartments and its morphogenesis over time in an efficient manner. An interesting approach of deriving expanded potential stem cells (EPSCs) from ESCs through a specific small molecule cocktail opened up a new line of research on embryogenesis. Taking hints from their name, EPSCs acquired an extended potential to self‐organize and mimic an eight‐cell stage embryo and further developed into morula‐like and blastocyst‐like structures, making it a suitable model for studying the early developmental signals and interactions involved in TE specification.^[^ ^23^ ^]^ Recently, mouse EPSCs were co‐cultured with mTSCs in inverted pyramidal microwells that, upon culturing in hypoxic conditions and exposed to blastoid‐inducing cues,^[^ ^15^ ^]^ gave rise to an extended potential blastoid (EPS blastoid) displaying some key features of a late blastocyst, such as the TE enveloped epiblast and PrE compartment polarized by a blastocoel cavity.^[^ ^24^ ^]^ The PrE further bifurcated into parietal (PaE) and visceral endoderm (VE)‐like cells, which were validated through the single‐cell transcriptomic data.

The approach of inducing primed ESCs (or mEpiSCs) to revert back to naïve state and further generating blastocyst‐like structures have also been explored recently in the mouse model namely the induced blastocyst‐like cysts (iBLCs).^[^ ^25^ ^]^ Although there has not been a lot of developmental progress yet when the different blastoid types were transplanted back into the uterus of foster mice, they could be explored further for in vivo implantation events such as decidualization and, invasion of the uterine wall by the trophoblast giant cells (TGCs). Such peri‐implantation embryo models would serve as a powerful tool in understanding the problem of implantation failure in humans.

#### 2.2.2 Mouse Peri‐Implantation Epiblast Rosettes

Aggregating naive mESCs within agarose hydrogel microwells and exposing them to a combination of chemically defined factors (cAMP, Retinoic acid, Chir, and Fgf4) induced the formation of an epiblast/PrE niche. In basic medium, this niche spontaneously progressed into a polarized epiblast epithelium with an extraembryonic endoderm‐like layer that both surrounded a pro‐amniotic cavity, reminiscent of the post‐implantation stage embryo. Single‐cell RNA analysis revealed that the primitive endoderm cells diverged into parietal‐like and visceral‐like extraembryonic endoderm precursors in the absence of trophoblastic tissues.^[^ ^22^ ^]^ The epiblast rosette is a model that allows to investigate the epiblast/PrE interactions and its coordinated progression toward other embryonic and extra‐embryonic tissues. An interesting finding using mouse epiblast models cultured within Matrigel was that the lumen formation in epiblast rosettes is driven by the exit from the naive pluripotency state and the secretion of sialomucins (Podxl) that drives the repulsion of polarized epiblast cells to give rise to a pro‐amniotic cavity at the center.^[^ ^26^ ^]^

#### 2.2.3 Egg‐Cylinder Models

Co‐culturing ESCs and TSC clumps with Matrigel gives rise to post‐implantation embryo‐like structures called ETS embryos, displaying some key features of embryo development, such as epiblast specification, lumenogenesis, and TE epithelialization.^[^ ^15^, ^27^ ^]^ The ETS embryos have been shown to form epiblast rosette‐like structures that develop a pro‐amniotic cavity via a nodal trigger from the epiblast with an adjacent extraembryonic ectoderm‐like compartment, resembling the egg‐cylinder of an implanted embryo. As such, this model could be beneficial in understanding the communication between the two niches to drive the formation of a pro‐amniotic cavity and the cross‐talk between the TE‐derived extra‐embryonic compartments on the specification of the axis in the epiblast. Moreover, upon BMP signaling, these embryos developed primordial germ cell (PGC)‐like structures at the site of posterior embryonic/extraembryonic junction, which has important potential for studying the microenvironmental cues regulating PGC development in vitro.

Post‐implantation egg cylinder‐like structures were also observed in the above‐mentioned EPS blastoids, which continued to develop epiblast rosettes with a lumen driven by the basement membrane secretion from the visceral endoderm layer surrounding it,^[^ ^25^ ^]^ and when co‐cultured with mTSCs, displayed an extraembryonic compartment abutting the epiblast proximally.^[^ ^24^ ^]^ Recently, it was shown that when mouse extraembryonic endoderm (mXEN) cells, which are derived from the PrE of a blastocyst, are combined with mESCs and mTSCs, they also undergo morphogenesis into post‐implantation embryo‐like structures, named ETX embryos.^[^ ^28^ ^]^ Upon differentiation, the XEN cells displayed a VE identity and secreted a basement membrane, thereby substituting the need for Matrigel to permit polarization and lumenogenesis of the pluripotent epiblast. These embryo models have been shown to undergo lumenogenesis and give rise to gastrulating tissue types, such as mesoderm and primordial germ cells, including the anterior visceral endoderm (AVE) that is responsible for the symmetry‐breaking event in mouse.^[^ ^29^, ^30^ ^]^ Despite that ETX embryos recapitulate some interesting features of post‐implantation embryo development, resolving further extra‐embryonic tissue types, such as the ectoplacental cone, TGCs and extraembryonic mesoderm (ExM) derivatives could be beneficial in understanding the post‐implantation morphogenesis better.

### 2.3 Mouse Gastrulation Models

Gastrulation is a process marked by a series of simultaneous morphogenetic events, such as symmetry breaking, germ layer specification, and patterning, reviewed in refs. ^[^ ^29^, ^30^, ^37^ ^]^. Despite the extensive knowledge of these events, there are still uncertainties regarding their exact mechanisms. In mouse, the development of gastruloids,^[^ ^14^, ^36^, ^38^, ^39^, ^40^ ^]^ which are formed by the self‐organization of mESCs through a Chir (Wnt/β‐Catenin agonist)/ActivinA exposure or a Chir pulse on 96‐well plates, generated 3D elongated structures expressing markers for neural, mesodermal, and endodermal cells.^[^ ^36^ ^]^ Further, embedding 96‐hour‐old gastruloids within a 10% Matrigel solution in culture medium induced somitogenesis.^[^ ^41^ ^]^ Matrigel has often been used as an extracellular matrix (ECM) substitute that accounts for the signals necessary to proceed development in vitro. These signals need to be further elucidated to better tailor the well‐defined chemical and mechanical properties of synthetic hydrogels and polymer platforms to facilitate embryonic development.

One of the recent gastruloid models, called Trunk‐Like Structures (or TLS), can induce somitogenesis and neural tube formation in the presence of a Wnt activator when embedded in 5% Matrigel at 96 h post aggregation, while most of the anterior patterning including AVE migration and heart formation as well as extraembryonic tissue derivatives are absent so far.^[^ ^42^ ^]^ Another interesting study using mESCs‐derived gastruloids generated beating heart progenitors through the exposure to cardiogenic factors, including Fgf, ascorbic acid, and VEGF (vascular endothelial growth factor), which demonstrated a fetal early cardiac‐like development with similar spatial and temporal patterning.^[^ ^43^ ^]^ The wide potential of mESCs to regulate the differentiation of advanced post‐implantation embryonic structures highlights the plasticity when cultured in vitro and thus, the challenge now lies in the further exploration of its ability to give rise to extraembryonic cell types.

### 2.4 Human Peri‐ and Post‐Implantation Models

In humans, the morphogenetic events driving embryonic development diverges from the mouse system significantly beyond the blastocyst stage. In addition, the challenges involved in the study of human embryogenesis have an added dimension of ethical concerns surrounding the development of human embryos in vitro for research. This emphasizes the relevance of stem cell‐based embryo models as a replacement for natural embryos to uncover the critical details that can accelerate advancing our knowledge on early human embryonic development.

Following implantation in humans, the epiblast and hypoblast compartment expand and form a bi‐laminar disc. The potential of human embryonic stem cells (hESCs) has been extensively researched to mimic some stages of human development (Table **2**). Human pluripotent stem cell (hPSC) aggregates embedded within a basement membrane‐like gel (Geltrex), later named PASE (Post‐implantation Amniotic Sac Embryoids), recapitulated the peri‐implantation amnion and displayed some of the molecular signatures of the natural embryo, including the induction of endogenous BMP signaling to drive lumenogenesis (Table 2).^[^ ^44^, ^45^ ^]^ The asymmetric human epiblast model mimics a 10‐day human epiblast by the aggregation of hESCs within a polymeric hydrogel coated with Matrigel and can break the symmetry with a uniform BMP4 signal.^[^ ^46^ ^]^ The information from these peri‐/post‐implantation epiblast models could be used to reproduce the features on a synthetic platform to decipher the different signaling and mechanical triggers required for their further development.

**Table 2:** The current human embryo models with the platform in which they are cultured, the major finding using that model, and the morphological stage of human development they represent

Embryo model	Culture platform	Significant finding	Stage of embryonic development they represent
PASE (hESCs)	3D ECM matrix mTeSR1 medium + Geltrex^[^ ^45^ ^]^	– Initial cell seeding density determines the type of epithelialization (columnar or squamous) in the ES cells, which in turn decides the amniotic‐like cells and epiblast‐like cells^[^ ^45^ ^]^ – Spontaneous pro‐amniotic cavity formation occurs via a BMP/SMAD pathway	Week 2–3
Asymmetric human epiblast (hESCs)	Polymeric hydrogel 3D matrix supplemented with matrigel/transwell filters^[^ ^46^ ^]^	– Embedding hESCs on a synthetic hydrogel supplemented with matrigel, resembles a pluripotent epiblast with an apico‐basal polarity^[^ ^46^ ^]^ – A varying concentration of BMP4 determines the fate of the epiblast cells whereby a 1 ng mL^−1^ of uniform BMP4 supply breaks the symmetry and induces Bra+ cells – The epiblast clusters start with uniform BMP4 signaling but later establish a concentration gradient of Wnt to break the symmetry	Day 10 of a human embryo
Micropatterned colonies (hESCs)	Circular micropatterned plates (1 cm) CYTOO chip^[^ ^47^, ^48^, ^51^ ^]^	– The number of cells, size of the micropatterned surface, and the signaling molecules they are exposed to affects the patterning of ESC colonies^[^ ^47^ ^]^ – hESCs on micropatterned surfaces displayed radial symmetry with an endoderm and mesoderm layer surrounded by a TE‐like layer – 2D surface enabled better imaging and signaling manipulation^[^ ^55^ ^]^	hESCs ≈day 18
Human gastruloids (hESCs)	Low adherence plates^[^ ^40^, ^52^, ^53^, ^54^ ^]^	– Culturing hESCs in Chiron 1 day before and after seeding onto plates leads to an elongation of the ESC clump and shows a posterior patterning of PS^[^ ^52^ ^]^ – Single‐cell transcriptomics data suggest signatures of somitogenesis in the gastruloids despite morphologically lacking anterior patterning	Day 5–6 to day 14

The development of hESCs on a micropatterned surface under a BMP4 influence generated organized colonies demonstrating radial symmetry with germ layers consisting of an outer TE, middle endoderm, and inner mesoderm/primitive streak (PS) layer.^[^ ^47^, ^48^, ^49^ ^]^ Transcriptional and morphogenetic profiling showed that extra‐embryonic ectoderm (ExE)‐like cells were generated within this model, which indicated the expression of TE and amnion specific genes.^[^ ^50^ ^]^ A recent study on this system revealed that the specification of all the three germ layers with a TE layer could be achieved with a timely exposure to Wnt, BMP, and/or Nodal and that there exists coordination between different signaling pathways to regulate the different tissue specification.^[^ ^51^ ^]^ Recently, hESCs grown as suspension cultures on non‐adherent plates with Chir, gave rise to compact elongated structures, called human gastruloids, that spatiotemporally resembled human gastrulation.^[^ ^40^, ^52^, ^53^, ^54^ ^]^

## 3 Substrate and Development

The timing of embryonic development is strictly regulated by the synchronous communication between different cells and between the cell and the surrounding microenvironment.^[^ ^56^, ^57^ ^]^ Stem cell‐based embryo models allow the dissection of mechanical (e.g., substrate or platform) and biochemical cues (e.g., signaling or morphogenetic regulators) to tune the differentiation of pluripotent stem cells into specialized tissue structures. Several recent reports have shown the correlation of substrate properties, such as stiffness,^[^ ^58^ ^]^ composition,^[^ ^59^ ^]^ and bioactivity and mechanical constraints, such as elongated cavities, micropatterned surfaces, and geometrical shapes, on morphogenesis (reviewed in ref. ^[^ ^60^ ^]^). To mimic cell tension and mechanical stimuli in vitro, different ECM substitutes and hydrogels have been employed in the 3D culture of cell aggregates to allow for a better recapitulation of the in vivo scenario and to complement the diffusion of soluble factors uniformly to continue development.

ECM substitutes, such as Matrigel, fibrin, gelatin, and collagen, have been increasingly used to culture embryo‐like structures for their ability to provide the necessary basement membrane proteins that are crucial for inducing lumenogenesis,^[^ ^26^, ^27^, ^61^ ^]^ germ layer specification,^[^ ^62^ ^]^ somitogenesis,^[^ ^41^, ^42^ ^]^ and neural patterning.^[^ ^18^, ^42^ ^]^ Polymeric hydrogels such as polyethylene glycol (PEG), polyacrylamide (PA), or agarose hydrogels of tunable stiffness and material properties have shown to recapitulate the morphology of the endometrial surface and provide the required mechanical cues to regulate pluripotency^[^ ^63^, ^64^, ^65^ ^]^ and differentiation of stem cells^[^ ^66^ ^]^ (reviewed in ref. ^[^ ^67^ ^]^), including the facilitation of post‐implantation embryonic development.^[^ ^22^, ^46^ ^]^ Besides providing mechanical support, modeling complex biological processes require dynamic matrices to recapitulate the in vivo system better. Recently, the development of advanced synthetic matrices (reviewed in ref. ^[^ ^68^ ^]^), such as functionalized host–guest supramolecular hydrogels,^[^ ^69^ ^]^ has been recognized as a versatile platform for investigating complex biological processes to account for the inherent dynamicity of such systems.

To mimic some of the mechanical triggers observed during embryonic development in vivo, physical constraints have been introduced during the growth of embryo structures to study the importance of such cues. For example, induction of symmetry breaking or germ layer specification in mouse and human ESCs can be observed in vitro by using micropatterned surfaces,^[^ ^47^, ^48^ ^]^ micro‐cavities of varying sizes,^[^ ^70^, ^71^ ^]^ or microfluidics^[^ ^45^ ^]^ to physically guide the morphogenesis of embryo structures. Thus, incorporating such physical and mechanical triggers, as well as accounting for the dynamicity of morphogenetic events, allows the mechanistic modulation of system parameters and recapitulates some of the complexities during natural developmental processes.

## 4 Gaps in Mouse Embryo Model Landscape

### 4.1 Modeling Blastomere‐Stage without Primary Tissues

**Figure 3:**
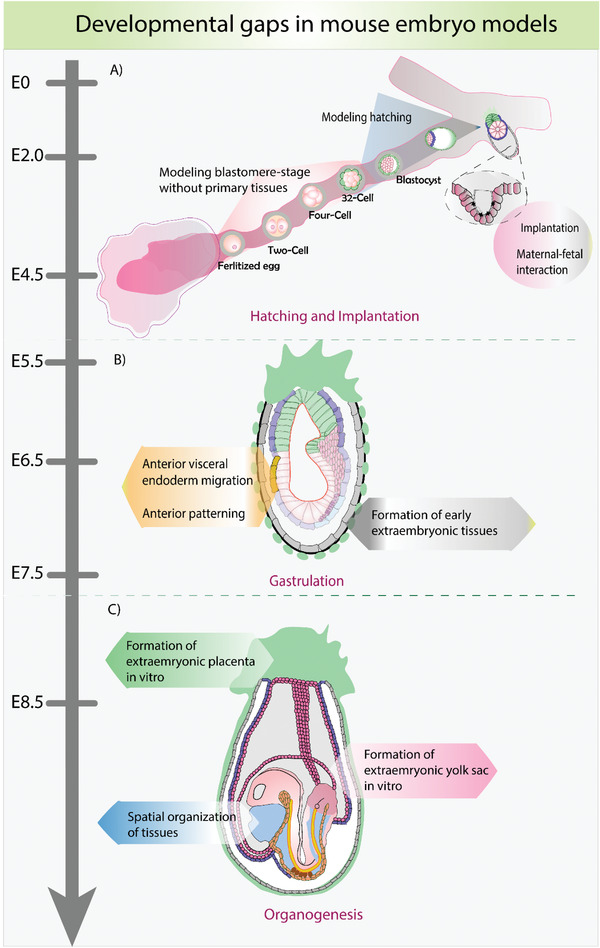
Schematic representing potential gaps in modeling mouse embryonic development in vitro. A) Modeling the blastomere stages of development could shine light on epigenetic imprinting, chromosomal aberrations, and plasticity during early stages. Modeling the process of hatching in vitro could help to further elucidate the role of mechanical factors and their implications on embryonic fate decisions. Opening the black‐box of embryonic implantation could shed some information on the biochemical and physical cues from mother to the fetus. B) The current embryo models lack some of the extraembryonic tissues such as parietal endoderm, Reichert's membrane and trophoblast giant cells, and anterior patterning events. C) Modeling the yolk sac derivatives, such as amnion, chorion, and allantois, still need to be resolved in the embryo models.

One of the challenging gaps in the study of early mammalian embryogenesis is the modeling of blastomere stage development in vitro. Embryonic development begins with the fertilization of the oocyte (egg cell) by a sperm cell, giving rise to a fused cell called zygote that undergoes subsequent cell divisions without expanding in size until the blastocyst stage (Figure 3). 2‐cell stage embryos are recognized to contain truly totipotent cells which can individually give rise to the entire conceptus.^[^ ^72^ ^]^ It has been observed over the years that in mice, the cell fate bias toward the embryonic or abembryonic axis is influenced—but not dictated—as early as from the 2‐cell stage by parameters including the sperm entry position and the plane of cleavage (reviewed in ref. ^[^ ^73^ ^]^). The potency of the cells from different stages of blastomere has been investigated extensively over the years.

It has been observed that the cells of the 8‐ or 16‐cell embryos are already pre‐destined to form either the ICM or TE lineage by their apical‐basal polarization and expression of key transcription factors, such as Cdx2 and Oct4.^[^ ^74^ ^]^ This is also evident when dissociated single cells from these stages do not make up the entire conceptus without being complemented with carrier blastomeres.^[^ ^75^ ^]^ Because of these observations, the definition of totipotency has been debated over the years and efforts have been made to extend pluripotency or induce totipotency in embryonic stem cells (reviewed in ref. ^[^ ^2^ ^]^). Recently, EPSCs derived from ESCs or somatic cells under the influence of specific signaling molecules (Fgf4, Bmp4, Chir, and A83‐01) self‐organize into structures that resemble an 8‐cell stage embryo.^[^ ^1^ ^]^ This model system could aid in our understanding of early cell fate specification and plasticity.

Another area that has been recently receiving a lot of attention, in a more clinical prospect, is the in vitro maturation of oocytes to produce follicular cells derived from pluripotent stem cells or primordial germ cells from embryos/embryo models^[^ ^76^ ^]^ (reviewed in refs. ^[^ ^77^, ^78^ ^]^), which when subjected to in vitro fertilization develop blastomeres. There has been a growing interest in exploiting gametogenesis in vitro to study blastomere development using stem cells, whereby oocytes ^[^ ^79^ ^]^ and sperm ^[^ ^80^ ^]^ cells derived from pluripotent stem cells can be used to form cleavage‐stage embryos. These studies would provide valuable information on the crucial first few stages of development in mice and other mammals and hold high significance for the human system in the case of fertility problems.

Interesting research from the previous decade showed the induction of parthenogenesis in mammalian haploid oocytes through an electric pulse or oocyte manipulation resulting in an embryo formed solely from egg cells,^[^ ^81^, ^82^, ^83^, ^84^ ^]^ but these embryos required primary tissues or tissue‐derived factors from the parent such as ovarian follicular cells, maternal fluids, or testicular cells to develop blastocysts and, in addition, had developmental defects at the later stage due to factors, such as genetic imprinting.

The current challenge is in recreating the early embryonic niche in vitro without the use of primary tissue material. This could be investigated by understanding the signaling and molecular factors involved in the interaction between the maternal tissue or follicular fluid and the in vitro‐derived (parthenogenetic) embryos. There have been various attempts to mimic the follicular microenvironment in vitro both as 2D substrates and 3D complexes that allowed follicle growth.^[^ ^85^ ^]^ These systems incorporate natural polymers, such as collagen, or synthetic polymers, such as PEG, to allow for the development of oocytes within the follicles. Such systems could be explored for their interaction with in vitro‐derived embryos to study the initial stages of development. Also, derivation of ovarian follicular cells from induced pluripotent stem cells (iPSCs) would allow large‐scale genetic manipulation to facilitate studying the genetic underpinnings of early embryonic events, such as zygotic genome activation, epigenetic imprinting, cleavage, and compaction.

In an alternative approach, oocytes were recently generated en‐large through the overexpression of a set of transcription factors in pluripotent stem cells. However, these in vitro‐derived oocytes are of yet developmentally incompetent.^[^ ^86^ ^]^ One of the major applications of parthenogenetic embryos could be to derive robust and clinically relevant parthenogenetic human^[^ ^87^ ^]^ and mouse embryonic stem cells.^[^ ^88^ ^]^ These ESCs could be used as a tool to look deeper into the function of specific genes in maternally inherited genetic disorders.

### 4.2 Formation of Extraembryonic Tissues

During implantation, the mouse embryo undergoes morphogenesis to embed itself in the mother's endometrium, thereby establishing pregnancy. This event is marked by the differentiation of PrE into PaE and VE, whereby the PaE cells migrate and distribute along the trophoblast giant cells that together secrete high amounts of ECM^[^ ^89^ ^]^ and form a Reichert's membrane.^[^ ^90^, ^91^, ^92^, ^93^ ^]^ This protective layer is crucial for establishing the initial contact of the conceptus with the uterine wall. Meanwhile, the VE extends throughout the embryonic and extraembryonic ectodermal compartment that is later involved in the anterior–posterior symmetry‐breaking event. Some of the extraembryonic tissues that develop synergistically with the embryonic tissues and serve important biochemical and mechanical roles are mentioned below.

#### 4.2.1 Parietal Endoderm

The mouse embryo models that capture the specification of the PrE currently lack the progression into post‐implantation parietal endoderm tissue and the dynamic formation of distinct types of visceral endoderm, which are crucial for the generation of the yolk sac and spatial coordination of the nascent epiblast. Among these models, the induced PrE/Epiblast rosettes ^[^ ^22^ ^]^ and EPS blastoids^[^ ^94^ ^]^ indicate the formation of PaE‐like cells flanking the epiblast compartment. However, most of the models are still yet to sustain these structures because of the lack of extra‐embryonic ectoderm and maternal tissues.

#### 4.2.2 Parietal Yolk Sac

An interesting event during implantation is the interaction of the parietal yolk sac, composed of PaE and TGCs separated by the Reichert's membrane, with maternal tissues. Parietal yolk sac is the outer layer of separation between the fetus and endometrium and provides a supportive niche for the further development of the embryo. Modeling parietal yolk sac interface with endometrial tissues on a bioengineered platform would provide some insights on the maternal–fetal communication as well as its role in the placental formation. This could be developed, for example, with the established differentiation protocols of PaE and TGC generation^[^ ^95^ ^]^ and co‐culturing them with maternal stromal or endothelial cells on a layer of hydrogel made of basement membrane components.

#### 4.2.3 Trophectoderm

Most of the current embryo models, despite lacking the extraembryonic derivatives, continue to progress toward post‐implantation structures. However, the shortcoming of these models is prominent in their restricted development when transplanted back into the mother. One of the most important reasons could be attributed to the lack of appropriate communication between the ESC and TSC compartment that prevents further progression in development. Such a gap could be investigated in vitro in three ways. The heterogeneity in the pluripotent state of the cell (along the continuum of toti‐/pluripotency including naive and primed ^[^ ^2^ ^]^), the mitotic cycle, or the cell number during the seeding could be some of the possible reasons. Sorting the cells based on their cell state and forming aggregates of similar cells could indicate the importance of cell state or shed light on the plasticity of these stem cells. A second way could be to observe the developmental progression of these embryo models in vitro by subjecting the embryo models to a large‐scale screening for different signaling molecules that aid in advancing the development of these models. A third way could be to study the interactions between the two compartments and their role in the developmental progression. This could be verified by decoupling the ESC and TSC compartments through a receptor knock‐out for one tissue type (e.g., E‐Cadherin knock‐out to restrict the formation of TE in the 8‐cell stage^[^ ^96^ ^]^) and following the development under different culture conditions.

#### 4.2.4 Fetal–Maternal Interface

Since maternal cues constantly drive morphogenesis in the embryo at different stages, combining endometrium cultures or recently established endometrial organoids^[^ ^97^ ^]^ with embryo models is an intriguing approach to further elucidate the underlying mechanisms. Such an organoid‐based system could provide a near‐physiological niche and can be incorporated with pre‐ and peri‐implantation embryo models to determine the complicated interactions in vitro. Alternatively, a synthetic endometrium composed of ECM components or synthetic polymer hydrogels that can mimic the chemical and mechanical properties of natural tissues could be a very powerful means to understand tissue remodeling and the biochemical/mechanical cues required to support embryonic development (reviewed in ref. ^[^ ^98^ ^]^).

### 4.3 Formation of the Extraembryonic Yolk Sac In Vitro

The extraembryonic (ExEm) tissues that form and support the future yolk sac or the placenta through the umbilical cord begin to appear in embryos around the time of gastrulation. The epiblast ingresses at the PS to give rise to ExM that later contributes to the amnion, allantois, and the chorion (Figure 3).^[^ ^99^, ^100^, ^101^ ^]^ The completion of the development of all these ExEm structures, by the formation of an exocoelomic membrane, is followed by the completion of gastrulation and the induction of somitogenesis.^[^ ^102^ ^]^

There has been some research on the development and function of these ExEm structures through the work on tissue explants and in vivo cell tracking.^[^ ^103^, ^104^ ^]^ Recently, the trajectory of ExM movement from the PS was visualized through two‐photon microscopy, and it was observed that a difference in the expression of specific cytoskeletal proteins occurs that mark the development of embryonic and extraembryonic mesoderm from the PS.^[^ ^105^ ^]^ However, there are still some major knowledge gaps in capturing the complete developmental progression, such as the morphogenesis of these structures in response to specific signals, the mechanism of its formation, and the interaction between different ExM derivatives.

This gap could be addressed, for example, through the derivation of extra‐embryonic mesodermal cells from the PS, where the anterior PS cells give rise to the embryonic mesoderm and the posterior PS cell migrate proximally to form the ExM. Alternatively, mESCs/mEpiSCs could be directed to differentiate into ExM cells through a specific exposure of, for example, BMP4 as discussed in ref. ^[^ ^106^ ^]^. These cells could be cultured in vitro to give rise to amnion, allantois, or the chorionic structures through the careful manipulation of signaling regulators. Such knowledge could then be transferred to embryo models to enable a simultaneous development of ExEm tissues from the PS. It would also be an informative strategy to generate ExM reporter lines, to track their development from the primitive streak and how they further contribute to the amnion and umbilical cord.

### 4.4 Formation of the Extra‐Embryonic Placenta In Vitro

The polar trophectoderm that abuts the epiblast gives rise to the extraembryonic ectoderm (ExE) and the ectoplacental cone, together contributing to the placenta. The current embryo models contribute more readily to the embryonic lineages, or more specifically to the posterior developmental programs, in comparison to extraembryonic tissues. It has been known that the ExE plays a role in the PS specification and AVE migration to establish symmetry breaking,^[^ ^107^ ^]^ but the importance of trophoblast derived tissues is more emphasized in their role of invading the maternal tissues at the time of implantation via the ectoplacental cone, thereby forming the fetal portion of placenta, connecting the mother and the fetus (Figure 3). Such an interface could be modeled in vitro using a microfluidic platform, for example, similar to a human placenta‐on‐a‐chip model (reviewed in ref. ^[^ ^108^ ^]^),^[^ ^109^ ^]^ incorporating mouse TSCs and maternal epithelial, stromal, and/or endothelial cells.

The umbilical cord of mice is derived from the ExM and it connects the fetus to the mother through the placenta, which is derived from the ExE. One of the interesting interactions that have not been explored yet in mice is the fetal‐umbilical cord‐placental interactions. With the promising improvement of microfluidic technology to produce robust and reliable 3D multicellular structures in specific microenvironments, such as endometrium‐on‐chip models,^[^ ^110^, ^111^ ^]^ there is hope in the translation of such technology to explore mammalian embryogenesis in a new light. For example, designing a microfluidic lumen‐based system by a sacrificial template technique in hydrogels lined with ExM cells, which are only separated by a thin hydrogel layer from an embryo model compartment or another tubular compartment lined with placental cells (derived from TSCs or placenta), could illuminate the nature of interactions between the fetus and the mother.^[^ ^112^, ^113^, ^114^ ^]^ A murine umbilical cord model in vitro is also relevant for the human scenario and can provide new perspectives on the fetal side interactions.

In another approach, mouse post‐implantation models that mimic gastrulation^[^ ^14^, ^28^, ^36^, ^39^ ^]^ could be explored further for the formation of ExEm tissues or precursors after the formation of the PS. Currently, these systems are not co‐regulated by the formation of ExEm tissues due to the absence of these cell types to begin with, for instance in gastruloids, or the potential to form ExEm tissues is lost through the specific signals they are exposed to. Further, the posterior patterning bias observed in these embryo models draws emphasis on the exploration of anterior signaling events in these models. This could be done, for example, by using microfluidic tools to establish temporally and locally constricted morphogen gradients in gastrulating embryo models to screen for the effect of different morphogens, such as Nodal or Wnt, on the ExEm cell fate specification.^[^ ^115^, ^116^ ^]^

### 4.5 AVE Migration

During implantation, the VE forms an epithelial layer that envelops the epiblast and ExE. The induction of PS at the posterior side of the epiblast triggers the migration of a specialized group of VE cells, called the distal visceral endoderm (DVE), toward the anterior side via signals from the ExE and epiblast.^[^ ^107^, ^117^, ^118^, ^119^, ^120^, ^121^, ^122^, ^123^, ^124^, ^125^ ^]^ This event is responsible for anteroposterior axis specification, including the patterning of head and heart, and for regulating the posterior signals (Figure 3).^[^ ^126^, ^127^ ^]^

Although the symmetry breaking event requires concerted efforts from all the compartments, recent work on stem cell‐based gastrulating embryo models shows that the signals from epiblast are sufficient to elicit axis specification and neural initiation, bypassing the anterior patterning events, namely the AVE induction and migration.^[^ ^11^, ^14^, ^38^, ^128^ ^]^ In models with mESCs, manipulation of biochemical signals such as Wnt and establishing a surface contact^[^ ^70^ ^]^ induced symmetry breaking. These findings highlight the plasticity of different cell types in the in vitro conditions and their potential to mimic different features of natural developmental processes. Such synthetic systems can be employed to study the extent to which they can further the development and if modulating different signaling components, such as Nodal and Wnt, which are important for the specification of PS and AVE, would give rise to endodermal structures more synchronous to the developmental timeline.

The AVE secretes Nodal antagonists, Cerberus‐like (Cerl), and Lefty, to regulate the posterior fate specification. Similarly, the ExE regulates Nodal signaling to restrict the AVE induction in the distal side and not with the proximal VE. These events can be observed using microfluidic tools, for example, to establish localized signaling centers or a partial gradient of soluble regulators such as retinoic acid (RA), Dickopf (Dkk1), Cerberus (Cer1), or noggin to visualize the morphological effects on the anterior and posterior patterning in gastruloids.^[^ ^116^, ^129^ ^]^ The use of the current embryo models to study the symmetry breaking from the perspective of the ExE would shed some light on the mechanism of induction and give clues about the further development of the embryo models.

## 5 Gaps in Human Embryo Models

### 5.1 Drawing Parallels between Mouse and Human Pre‐Implantation Development

Due to its similarity and relative simplicity in development, mouse models have long been used as a system to understand early human embryogenesis up to pre‐implantation stage.^[^ ^130^, ^131^ ^]^ Human embryogenesis, however, diverges from the mouse system at the implantation stage, where it forms a bilaminar disc with the epiblast and hypoblast compartments, that later engulfs the embryo with an amnion‐yolk sac layer. Beyond morphology, zygotic genome activation, blastocyst cell fate specification, and signaling programs are different in the two models.

There has been huge progress in the study of early human embryogenesis with the use of human embryos and with the clues from other vertebrate model systems. Recently, the derivation and maintenance of hESCs^[^ ^132^, ^133^ ^]^ and iPSCs have paved the way for generating 2D and 3D stem cell‐based embryo models on a bioengineered platform under defined conditions. The current human embryo models only capture snapshots of key features of peri‐^[^ ^46^ ^]^ and post‐implantation stages^[^ ^45^, ^46^, ^52^, ^134^, ^135^ ^]^ of development, such as early amniogenesis, symmetry breaking, and germ layer specification.

**Figure 4:**
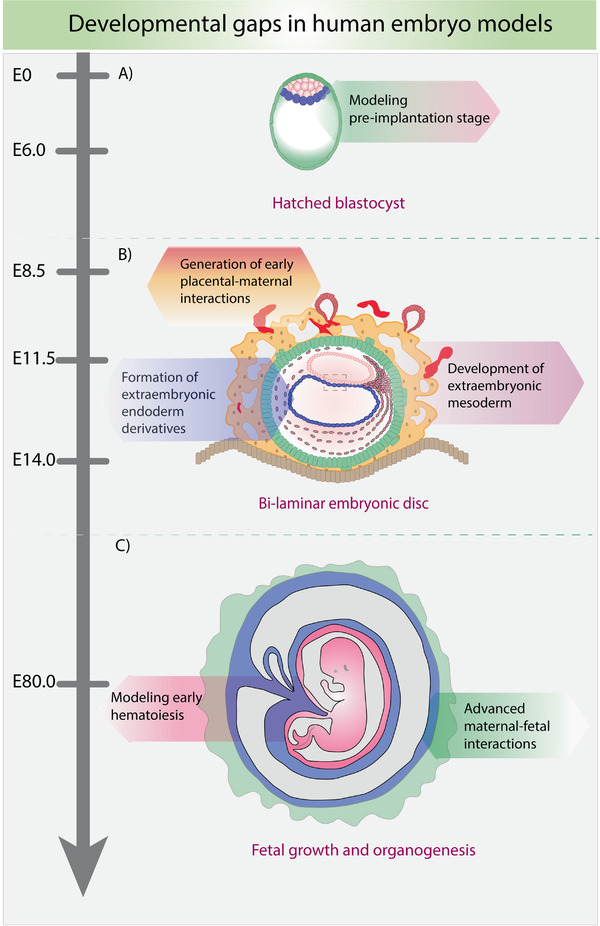
Schematic representing potential gaps in modeling mouse embryonic development in vitro. A) Modeling the pre‐implantation stage of development could provide valuable information on the signaling cues governing cell fate decisions and morphogenesis. B) The lack of sufficient information on the formation and derivation of extraembryonic mesoderm could be addressed by the differentiation of these cells in vitro. The interaction between different types of trophoblast cells and their invasion of the maternal endometrium could be modeled to better understand the mechanisms behind this process, which could be very useful in the case of implantation failures and fertility‐related problems. Formation of extraembryonic endoderm derivatives includes the hypoblast‐derived cells that later contribute to the yolk sac and extraembryonic mesoderm. C) Modeling early fetal hematopoiesis, which begins at the yolk sac, could help in the diagnostic testing of fetal blood supply for diseases during development.

However, there are some morphogenetic gaps in the developmental timeline of these embryo models that are yet to be ascertained (Figure 4). For example, the in vitro human pre‐ and peri‐implantation models have not been established like for the mouse system (Figure 4). One of the reasons could be attributed to the state of the human PSCs that have been used so far to obtain 3D structures. The human ESCs and iPSCs that have been conventionally established are transcriptionally similar to a primed or post‐implantation epiblast^[^ ^13^, ^136^, ^137^ ^]^ and therefore have limited extraembryonic potential. The recent derivation and long‐term maintenance of naïve PSCs^[^ ^136^, ^138^, ^139^, ^140^ ^]^ have opened up new possibilities for exploring the plasticity and potential of these cells to give rise to differentiated tissue types.

Another reason could be attributed to the lack of sufficient information on the pathways regulating the specification of ICM, TE, and PrE cell fate. Recently it has been found that conserved mechanisms specify the TE in mouse and human.^[^ ^21^, ^141^, ^142^ ^]^ From the 8‐cell stage, the outer cells via the asymmetrical inheritance of keratin filaments^[^ ^143^, ^144^ ^]^ acquire an apical‐basal cell polarity with atypical protein kinase C expression at the peripheral cell membrane, nuclear Yap1, and restricted expression of TE factors.^[^ ^21^ ^]^ However, in contrast to mouse, it takes in human until the blastocyst stage to restrict plasticity in TE cells, due to retention of pluripotency genes such as Oct4 and Sox2.^[^ ^142^, ^143^ ^]^ The workings and functions of these plasticity differences between human and mouse are still yet to be elucidated.

The derivation of stem cells from the three compartments of a blastocyst, namely the hESCs, the hTSCs, and naive human extraembryonic endoderm cells (h END),^[^ ^145^ ^]^ has opened the possibility of studying the interaction between different cell types and the extent of developmental potential showcased by their self‐organization in 3D, for example, with a comparison to the single‐cell RNA seq data of human blastocysts.^[^ ^146^ ^]^ An obvious inspiration can be drawn from the mouse blastocyst models,^[^ ^15^, ^24^, ^25^, ^147^ ^]^ where hESCs or hEPSCs could be co‐cultured with hTSCs and/or hEND cells to inform on the signaling crosstalk and specification cues of the different lineages in the human blastocyst. Understanding the specific events in early embryonic development is especially important to get a better picture of the chromosomal aberrations in embryos,^[^ ^148^, ^149^ ^]^ implantation failures after in vitro fertilization, and pre‐implantation genetic testing.^[^ ^150^ ^]^ The use of hPSCs with mutations for genetic disorders^[^ ^151^, ^152^ ^]^ in these models, combined with high‐throughput screening for its developmental potential, could provide valuable information that is otherwise difficult to obtain from human embryos.

The other parameter required for co‐culturing the stem cells in vitro, apart from the culture conditions and cell types, would be to identify the suitable platform required for developing embryonic structures. Similar to the mouse embryo models, the hESCs and hTSCs could be cultured as either free‐floating entities on a non‐adherent surface or in agarose microwells,^[^ ^14^, ^15^ ^]^ on ECM coatings,^[^ ^20^ ^]^ or embedded in ECM matrices.^[^ ^27^ ^]^ Along this line, microfluidic platforms, such as combinations of microwell arrays and single‐cell dispensing units or mix and sort droplet microfluidics, could be designed to manipulate the system for generating embryo models with the desired cell number, desired size, and controlled distribution of morphogens that would produce more robust embryo models.^[^ ^153^ ^]^

### 5.2 Formation of Extraembryonic Endoderm Derivatives

The extraembryonic endodermal tissues that begin at the end of the first week of development as hypoblast later establish the physical communication between the fetus and the mother to allow the transport of several biochemical molecules. During implantation, the hypoblast that lines the epiblast expands, and along with ExM, forms a disc, called the primary yolk sac, that lies below the amnion.^[^ ^154^ ^]^ The primary yolk sac is later replaced with a definitive yolk sac that has been known for being the primitive source of hematopoiesis and vascularization in embryos.^[^ ^155^, ^156^, ^157^ ^]^ The current research on early human embryogenesis focuses primarily on the development of embryonic tissues utilizing the self‐organizing properties of hESCs. The potential derivation and recapitulation of the different extraembryonic endodermal tissues using the available state‐of‐the‐art bioengineering tools and different cell types are discussed below (Figure 4).

#### 5.2.1 Hypoblast

Recently, naive human extraembryonic endoderm cells (hEND) have been derived from naive human ESCs by exposing them to Nodal, Wnt, and LIF Signaling molecules.^[^ ^145^, ^158^ ^]^ This has opened up new possibilities for investigating the differentiation potential of these hypoblast‐like cells into yolk sac and ExM tissues in a 3D microenvironment. There is still limited knowledge on the development of different ExEm structures and their interaction with the epiblast.

In mouse, chemically inducing the differentiation of primitive endoderm (PrE) in mESC aggregates led to the formation of post‐implantation epiblast rosettes with a cavity surrounded by VE.^[^ ^22^ ^]^ Combining the knowledge from the derivation of hEND cells from hESCs, together with the single‐cell transcriptomic data on human embryos, hESC aggregates could be modulated, for example, to allow progression into peri‐implantation epiblast‐hypoblast structures resembling a bilaminar disc. These structures could be developed on bioengineered platforms such as hydrogel microwells,^[^ ^22^ ^]^ micropatterned surfaces,^[^ ^159^ ^]^ or ECM scaffolds, that can confer a spatio‐temporal distribution of morphogens to allow for the interaction between different compartments. Understanding the mechanism behind the cellular patterning of ICM into epiblast and hypoblast could provide invaluable information on cell fate specification in human embryos.

#### 5.2.2 Yolk Sac Hematopoiesis

The hematopoiesis in humans is initiated in the yolk sac until the first three weeks of gestation and with the development of cardiac tissues, a source of local blood production occurs in the cardiac cavity. Gradually, the heart contractions allow the establishment of a fetal‐vitelline circulation^[^ ^160^, ^161^ ^]^ and later a definitive hematopoiesis occurs in different organs. There has been a long debate on whether the ExM, where blood formation occurs, is of epiblast or hypoblast origin. The potential of hPSCs (hESCs and hiPSCs) to generate hematopoietic progenitors and hematopoietic cell types may provide clues on the involvement of epiblast‐derived ExM in the initial blood formation.^[^ ^160^ ^]^ With the recent derivation of hEND from naïve hESCs through the activation of Wnt and Nodal,^[^ ^145^, ^158^ ^]^ new possibilities to investigate the source of early hematopoiesis have been recognized.

The current derivation of hematopoietic cell types from hESCs recapitulates some of the key developmental programs of primitive (from yolk sac) and definitive hematopoiesis (from other organs), although there exists heterogeneity in the generation of the different cell types when cultured in vitro. For example, some of the hESC‐derived blood cells still resemble the developmental programs of primitive hematopoiesis. Hence, an in vitro model to recapitulate the early erythropoiesis and hematopoiesis from the yolk sac could give us a better understanding of all the molecular players involved in hematopoietic specification and in the transition of the primitive to definitive blood source. One way to model this could be to utilize the human ESCs and hEND cells for mimicking the fetal yolk sac‐mesoderm using a hydrogel‐based co‐culture platform similar to a scaffold‐based multilayered endometrium‐on‐chip for instance.^[^ ^162^ ^]^ Such systems can have applications in the development of diagnostic strategies that detect fetal blood circulation and have clinical relevance in cellular therapies.

#### 5.2.3 Umbilical Cord

Another important organ formed during the third week of development is the umbilical cord that extends from the allantois along with the vascular network to form a tube connecting the fetus to the mother through the placenta. The umbilical cord forms a portal that allows the exchange of nutrients, waste, and gas via a feto‐maternal blood supply. Modeling an in vitro umbilical cord on a microfluidic platform, similar to the model explained above for mouse, through a directed differentiation of the ExM cells lining the tubular surface of a synthetic or natural hydrogel mimicking the in vivo vascularization network would be an interesting approach to investigate fetal‐maternal communication. The exchange of blood and nutrients, maintenance of the temperature, and the release of hormones could be some of the read‐outs that could be explored in such synthetic systems.

### 5.3 Generation of Early Human Placental–Maternal Interactions

In addition to the yolk sac, successful and healthy development of the embryo requires the simultaneous development of the placenta. Trophoblast cells from the TE invade the maternal endometrium during implantation and gradually form a villous network called the extravillous trophoblast (EVT) with the maternal blood vessels.^[^ ^163^ ^]^ The TE in contact with the deciduae, cytotrophoblast (CTB), forms a continuous layer surrounding the embryo while the syncytiotrophoblast (STB) establishes a stable maternal‐fetal exchange.^[^ ^164^, ^165^ ^]^ Due to the technical and ethical limitations, there is an increasing emphasis on the establishment of a trophoblast model in vitro to answer some of the questions surrounding its development and function (reviewed in detail in ^[^ ^166^ ^]^).

Recently, human trophoblast stem cells (hTSCs), derived from naïve hESCs and/or human placenta and from iPSCs,^[^ ^145^, ^167^, ^168^, ^169^, ^170^, ^171^ ^]^ have been established in vitro. Trophoblast cells from the first‐trimester placenta have been cultured in Matrigel droplets in a specified medium cocktail (containing EGF, FGF2, Chir, R‐Spondin, and A83‐01), which showed similar transcriptomic profiles as the first‐trimester placental cytotrophoblasts and developed EVT and SCT‐like structures.^[^ ^168^, ^172^ ^]^ However, these organoids were spatially oriented with an outer CTB and an inner STB layer in contrast to the natural scenario. The trophoblast organoids could be alternatively cultured on, for example, a microfluidic platform that can establish a gradient of Wnt activators/inhibitors to allow the simultaneous culturing of the three types of TSCs and promote a directed invasion of the EVT.^[^ ^44^, ^168^ ^]^

The derivation of TSCs from the early placenta is also ethically challenging. Hence, there is increasing hope for the development of robust hTSCs from the blastocyst, hESCs,^[^ ^169^, ^170^ ^]^ or somatic cell reprogramming^[^ ^171^ ^]^ that is well‐characterized and recapitulates the developmental potential of natural embryos. The potency of these cells and their differentiation could be thoroughly studied in scalable and highly parallelized microfluidic cell culture platforms, e.g. by systematic screening of libraries of small molecules, cytokines, or growth factors.^[^ ^173^ ^]^ These cells can be used to further improve the functionality of such trophoblast organoids for various applications.

The other interesting potential of trophoblast organoids is to investigate the placental interactions with maternal tissues and to study the complex maternal‐fetal barrier in vitro. Recently, several groups have developed a placenta‐on‐a‐chip model that incorporates hTSCs and fetal endothelial cells on a microfluidic platform.^[^ ^109^, ^174^, ^175^, ^176^, ^177^ ^]^ These models use either BeWo carcinoma‐derived TSCs or primary TSCs from the early placenta. It would be interesting to observe the functionality of the villous network by initiating vascularization in these tissues with a source of fetal blood supply from hematopoietic stem cells. This system could be applied as a proof‐of‐concept for understanding disease transmission from mother to the fetus, the effect of drugs, nutrition as well as in genetic abnormalities.

### 5.4 Development of Extraembryonic Mesoderm (ExM)

ExM is an important feature of the human implantation event and has been observed to be formed before the initiation of gastrulation, along with the formation of the primary yolk sac.^[^ ^178^, ^179^ ^]^ This is in contrast to the mouse where the ExM is specified after the formation of the mesoderm from the PS during gastrulation.^[^ ^180^ ^]^ In both cases, the ExM forms the major part of the yolk sac, amnion, allantois, and chorion. Although the origin of the ExM is known to occur at the peri‐implantation stage, its exact source and mechanism are still ambiguous (reviewed in refs. ^[^ ^156^, ^181^ ^]^). The observations in rhesus monkey embryos^[^ ^182^, ^183^, ^184^ ^]^ showed evidence of the origin of the ExM to be from hypoblast cells rather than CTB due to the presence of a basal lamina separating the CTB from the newly formed mesenchymal cells.

Recently, single‐cell transcriptome data from 3D cultured human embryos from day 6 to day 14 identified a unique group of T/Brachyury (mesodermal marker) expressing cells migrating from the epiblast and occupying the space between EPI and VE.^[^ ^135^ ^]^ This group of cells expressed Gata6, which has been a known marker for the hypoblast/yolk sac. This data is in contrast to the single‐cell RNA seq data from in vitro cultured cynomolgus monkey embryos, which showed that the ExM cells express Gata6 and Gata4 but not T.^[^ ^185^ ^]^ This kind of variation in the expression of a similar tissue sparks curiosity on the mechanisms behind this tissue type.

This is an important gap, mainly because of the scarcity of knowledge behind this important tissue type that contributes primarily to the yolk sac tissues and its unknown influence on the establishment of anteroposterior axis and gastrulation. So far, such studies in humans were challenging due to the delicate interval in which these events occur. With the derivation of stem cells of epiblast, hypoblast, and trophoblast origin, it is now possible to direct the differentiation of extraembryonic cell types, for example, with the induction of ExM lineage from hESCs through an exposure of BMP in the absence of Fgf2.^[^ ^106^ ^]^

A way to explore these features could be to make use of stem cell‐based embryo models such as human gastruloids^[^ ^38^, ^40^, ^134^ ^]^ formed from the self‐organization of hESCs in response to morphogens, such as BMP and Wnt, on bioengineered surfaces. The identification of a suitable marker for ExM or validating the corresponding marker from other organisms, such as mouse Flk1, would be an important step in visualizing and tracing the lineage specification. Also, recent technologies, for example, spatial transcriptomics, could be employed on 3D cultured embryos to find the origin of extraembryonic tissue types based on the clustering of different tissue groups.^[^ ^186^ ^]^

## 6 Conclusions and Future Perspectives

The last decade saw some remarkable advances in the field of stem cell‐based embryo models. These embryo models have helped in providing a better understanding of the processes involved in early mammalian embryogenesis such as the role of different morphogens in the specification of epiblast‐TE‐PrE in the early blastocyst,^[^ ^15^, ^22^ ^]^ the importance of mechanical and biochemical cues in the germ layer specification,^[^ ^45^, ^47^, ^187^ ^]^ and the plasticity of ESCs in giving rise to the different tissues of embryonic and extraembryonic origin. The derivation of stem cells from the blastocyst stage has enabled the genetic manipulation of different cells and following their behavior on 2D or 3D platforms has provided some clues on their possible characteristics in vivo.

However, the culture of a rather complex biological phenomenon in vitro comes with its limitations on how well these structures recapitulate the developmental timeline of the embryo. Most of the current embryo models are grown on static platforms using polymer matrices and designs, which only permit limited cellular remodeling of the microenvironment and dynamic control of cell culture conditions. A 3D cell culture platform with fixed geometrical constraints or material properties (e.g., stiffness), is predestined to only allow capturing of snapshots in development. This is an oversimplified imitation of a highly intricate in vivo scenario where the extracellular matrix is constantly remodeled, concentrations of morphogens, growth factors, and cytokines are continuously modulated, and dynamic interaction with the maternal environment results in synchronized embryo development.

On the one hand, recent developments of new advanced materials, such as dynamic hydrogels^[^ ^188^, ^189^ ^]^ that can change physical properties in response to external cues, and microfluidic systems that can overcome diffusion limitations in 3D cellular constructs and can be used to controllably enrich or deplete media over time in a spatiotemporally controlled manner^[^ ^61^, ^190^, ^191^, ^192^ ^]^ are promising steps toward programmable and intelligent cell culture systems. There will be a growing need for new precision tools to compensate on a technical level for missing cells and tissues that usually serve as important signaling centers and organizers in embryos to facilitate and maintain continuous development. However, there is a delicate balance between the complexity of these systems, for example, to initiate and properly guide cellular self‐organization in embryo models, and the biological relevance of obtained data. The critical reflection on this is becoming more and more important especially where benchmarking with the in vivo situation is strictly limited due to legal and ethical constraints. On the other hand, in many cases, it is not known yet what the roadblocks are that need to be overcome in order to unlock a continuous and sustained development in vitro. In addition, it is evident from the limited availability of human embryo models that further efforts have to be put in this field to improve our knowledge of early human embryogenesis, identify means to expand the repertoire of human embryo models and, capacitate them as relevant tools for clinical applications and reproductive health.

Another aspect to the development of embryo models is to assess their functionality to undergo morphogenesis when it interacts with maternal tissues, which is especially relevant in the case of humans. The current mouse blastoids recapitulate most of the developmental profiles of a natural blastocyst, including the transcriptional signatures. However, they still fail to proceed development when transplanted back into suitable mice despite eliciting the initial decidualization. One of the reasons could be attributed to the lack of concerted actions of mechanical forces, for example, caused by the hatching from the zona pellucida, which in vivo is well‐coordinated with implantation. The effect of such stimuli could be further investigated in vitro through imposing defined mechanical stress on the embryo model by using micro‐engineered mechanical barriers^[^ ^57^ ^]^ and controlled hydrostatic or hydrodynamic forces to emulate hatching in vitro. Further, designing novel multiplexed microfluidic assays and large‐scale, high‐throughput screening platforms compatible with the embryo models will help to learn more about how biological and environmental factors affect development.^[^ ^193^ ^]^

The success of synthetic embryology also depends on the development of new imaging techniques, such as 4D live cell imaging^[^ ^194^ ^]^ or light sheet microscopy, and how well these imaging techniques are compatible with the increasing complexity of the engineered microenvironments. Computational resources to validate the findings of the in vitro system to the in vivo scenario and computational modeling have already taken an important position in ensuring the progress of this field to keep up with new technologies. State‐of‐the‐art techniques such as single‐cell and/or spatial transcriptomics,^[^ ^41^ ^]^ tomo seq,^[^ ^195^ ^]^ de novo lineage tracing,^[^ ^196^ ^]^ and spatial mapping of single‐cell RNA seq data^[^ ^197^ ^]^ can help in streamlining the data obtained from different in vitro culture systems and extrapolate relevant information on specific cellular events or tissues that are otherwise difficult to observe morphologically.^[^ ^135^ ^]^

With constant improvement in the development of new models to represent specific features of embryonic development and growing knowledge on single‐cell data from in vitro embryos, the field of synthetic embryology is rapidly moving forward. Recognizing the gaps and identifying limitations of a system is important in gaining a full perspective on the applications of such systems and together, help in understanding human embryology, in‐vitro fertilization, developmental disorders, and other fertility related problems. By mapping the timeline of synthetic embryos, identifying some of the gaps, and providing potential ways to expand or develop new embryo models, this progress report aims at contributing to a better understanding of the current embryo model landscape and to evolve the field from modeling developmental snapshots to modeling embryogenesis.

## Conflict of Interest

The authors declare no conflict of interest.
